# Prevalence and associated factors of cognitive impairment among the elderly population: A nationwide cross-sectional study in China

**DOI:** 10.3389/fpubh.2022.1032666

**Published:** 2022-11-17

**Authors:** Feng Qin, Min Luo, Yang Xiong, Ni Zhang, Yanping Dai, Weihong Kuang, Xiaobo Cen

**Affiliations:** ^1^National Chengdu Center for Safety Evaluation of Drugs, State Key Laboratory of Biotherapy/Collaborative Innovation Center for Biotherapy, West China Hospital, Sichuan University, Chengdu, China; ^2^Department of Urology, West China Hospital, Sichuan University, Chengdu, China; ^3^Department of Psychiatry, West China Hospital, Sichuan University, Chengdu, China; ^4^Department of Nuclear Medicine, West China Hospital, Sichuan University, Chengdu, China

**Keywords:** prevalence, risk factors, Chinese elderly population, dementia, cognitive impairment

## Abstract

**Background:**

Cognitive impairments are associated with increased risk for progression to dementia. In China, limited surveys have been conducted to estimate the national prevalence and risk factors associated with cognitive impairment in China. This study aims to assess the national prevalence and modifiable risk factors for cognitive impairments in the Chinese elderly population.

**Methods:**

This cross-sectional study was based on the 2018 China Health and Retirement Longitudinal Study. The Mini Mental State Examination (MMSE) is recommended to test for cognitive impairment. Univariate and multivariate logistic regression models were used in assessing risk factors for cognitive impairments in the Chinese elderly population.

**Results:**

A total of 3768 participants aged 60 years or older were enrolled in this study. The national prevalence of cognitive impairments was 22.24% in China, and the prevalence of cognitive impairment was higher in the south-west region than in the north region (29.94 vs. 16.53%, *p* < 0.05). The risk for cognitive impairments was higher in the following participants: not married or not living with spouse relative to married with spouse present (OR = 1.39, 95% CI, 1.15–1.70; *p* = 0.001), nap duration of ≥ 90 min relative to 30–60 min (OR = 1.54, 95% CI, 1.20–1.98; *p* = 0.001), sleep duration of ≥ 8 h relative to 6–8 h (OR = 1.73, 95% CI, 1.29–2.31; *p* < 0.001), and depression relative to no depression (OR = 1.67, 95% CI, 1.41–1.97; *p* < 0.001). The risk of cognitive impairment was lower in participants living in the urban areas relative to the rural areas (OR = 0.57, 95% CI, 0.47–0.69; *p* < 0.001) and consuming alcohol once a month relative to never consuming alcohol (OR = 0.69, 95% CI, 0.51–0.94; *p* = 0.02).

**Conclusion:**

Cognitive impairment prevalence was high in the Chinese elderly population. The potentially modifiable risk factors for cognitive impairment should be further assessed in the development of interventions for the elderly Chinese population.

## Background

Dementia is arguably the most feared and devastating disease affecting the elderly population; and is a leading cause of disability and dependence of aged individuals, worldwide (1). According to the World Alzheimer Report, in 2019, more than 50 million individuals suffered from dementia globally, and this number is estimated to surge to 152 million by 2050 (2). Cognitive impairment is associated with increased risk of disability, increased health expenditures, and progression to dementia (3). Problems in memory, language, thoughts, and judgment are more prominent than normal age-related changes in patients with cognitive impairments, although the basic daily activities are not disrupted (4). Cognitive impairment ranges from mild to severe, and is one of the most common and disabling non-motor symptoms in elderly individuals. Mild cognitive impairment (MCI) is also known as cognitive impairment without dementia. It is considered a preclinical transitional stage between healthy aging and dementia, and gradually progresses to dementia in nearly 10–30% of patients with MCI (5). The updated estimated prevalence of MCI is 15.5%, and the prevalence of severe cognitive impairment (dementia) is approximately 6.0% in individuals aged 60 years or older in China (6). Notably, no effective medication is currently available for the treatment of cognitive impairment. Therefore, identifying the etiologies of cognitive impairments and suppressing the incidence are more important than treating them following their onset.

In the past decades, rapid demographic and epidemiological transition, have led to an aging population in China. Thus, the country has a large number of people with cognitive impairments. Many studies have investigated the prevalence and risk factors for cognitive impairments in China's general population (6–10). Older age, being female, rural residence, illiteracy, living without a partner, smoking, hypertension, hyperlipidemia, diabetes, heart disease, and cerebrovascular disease are the main risk factors for dementia and MCI (6–10). However, the prevalence and risk factors are vary among cities or regions of China, and limited surveys have been conducted to estimate the national prevalence and risk factors associated with cognitive impairments. These inconsistencies require further study for a realistic estimate.

In the present study, by conducting a nationwide cross-sectional survey, we aimed to estimate the prevalence of cognitive impairments and its modifiable risk factors in the elderly in China. The findings can help to enhance understanding of cognitive impairments and strategies for protecting the elderly population against cognitive decline.

## Methods

### Study sample and data cleansing

To investigate the prevalence of cognitive impairments in the elderly, the China Health and Retirement Longitudinal Study (CHARLS) 2018 follow-up dataset was downloaded and analyzed. The CHARLS was hosted by Peking University and approved by the Peking University Ethics Review Committee (IRB00001052-13074). Initiated in 2008, the CHARLS uses probabilities proportional to the size method to sample aging populations aged 45 years and above in the whole of China, with follow-ups in 2011, 2013, 2015, and 2018. The data were obtained from 150 counties and 450 villages in 28 provinces. The dataset from CHARLS are representative and of high quality. Detailed description and specific study design of the CHARLS project can be accessed from previous publications (11, 12) or its official website (http://charls.pku.edu.cn/). In the 2018 survey, the cognitive functions of participants aged 60 years and above were evaluated with the Mini-Mental State Examination (MMSE) questionnaire (13, 14). The flowchart of data cleansing is displayed in Supplementary Figure 1. In the present study, a total of 3,768 participants were analyzed.

### Assessment of cognitive function

Cognitive functions were assessed using the MMSE questionnaire, which comprises 30 items with scores ranging from 0 to 30. The questionnaire is widely used in epidemiology for cognitive function assessment. The MMSE consists of seven aspects: orientation to time (five items), orientation to place (five items), registration (three items), attention and calculation (five items), recall (three items), and language (nine items). Assessment was performed by two well-trained staff through a face-to-face interview with the native dialect. According to previous studies (10, 13–16), the cutoff of MMSE was set at 16/17 for illiterate individuals, 19/20 for individuals with 1–6 years of education, and 23/24 for individuals with at least 7 years of education. An MMSE score lower than the above-described cutoff values indicated cognitive impairment.

### Covariates

Covariates, including individual characteristics and medical histories, were investigated. Individual characteristics self-reported by the respondents (17) included age (60–70, 70–80, or >80 years), gender (male or female), marital status (married with spouse present vs. divorced/separated/widowed/married but not living with spouse), educational level (illiterate, elementary school, middle school, high school, or college degree and above), settlement (central of city/town, urban–rural integration zone, or rural), cigarette consumption (yes or no), alcohol consumption (more than once a month, less than once a month, or never); afternoon nap duration ( ≤30, 30–60, 60–90, and ≥90 min) (17, 18) and sleep duration ( ≤6, 6–8, and ≥8 h) (17–19) were used according to previous studies. Medical histories included information on depression (yes/no), hypertension (yes/no), diabetes (yes/no), arthritis (yes/no), digestive diseases excluding cancers (yes/no), kidney diseases excluding cancers (yes/no), liver diseases excluding cancers, and fatty liver (yes/no), and stroke (yes/no).

Depression was assessed using the Center for Epidemiological Studies Depression Scale-10 questionnaire (14, 20). Medical histories were mainly based on the self-reports of the respondents. Given differences in living and cultural habits, the living localities of the participants were categorized into six regions in the same manner as previous studies did (17, 21): north (Shanxi, Hebei, Beijing, Tianjin, and Inner Mongolia), northeast (Jilin, Liaoning, and Heilongjiang), east (Jiangsu, Fujian, Shanghai, Shandong, Zhejiang, Jiangxi, and Anhui), northwest (Qinghai, Shanxi, Xinjiang, and Gansu), southwest (Sichuan, Chongqing, Yunnan, and Guizhou), and south-central (Hunan, Henan, Guangdong, Hubei, and Guangxi).

### Statistical analysis

Data of clinical characteristics were summarized as proportions (%) according to data type. Descriptive statistics were used to investigate the prevalence of cognitive impairments in different groups. Moreover, univariate binary logistic regression was used in scanning risk factors for cognitive impairments. Then, correlation coefficients were calculated using Spearman tests to prevent collinearity before multivariate regressions were performed. The correlations among the covariates were low (Pearson correlation coefficients < 0.5, Supplementary Figure 2), and thus no variable was deleted (22). Variables with *p* of < 0.05 in univariate logistic regression were included in multivariate logistic regression analysis. Therefore, age, marital status, residence, alcohol consumption, afternoon napping, sleep duration, depression and liver disease were included in the multivariable logistic regression model. All the analyses and figures were made using R 4.0.1 (R Foundation for Statistical Computing, Vienna, Austria). A *p-*value of < 0.05 (two-sided) indicated statistical significance.

## Results

### Baseline characteristics and prevalence in the grouped population

Between July 2018 and March 2019, a total of 19,816 adults (≥ 45 years) were invited to participate in the CHARLS 2018 survey. First, 8,998 participants under 60 years of age were excluded. Then, 7,050 participants were further removed because the respondents refused to receive MMSE examination and their MMSE questionnaires had missing values. Finally, 3,768 (≥ 60 years) were enrolled (Supplementary Figure 1). The descriptive statistics of enrolled participants are listed in Table 1. The overall prevalence of cognitive impairment was 22.24% (95% CI, 20.94–23.60). In total, 3,768 participants comprised 2,451 (65.0%) males and 1,317 (35.0%) females, and 81.05% of the participants were married. About 30.8% of people lived in urban areas, 60.7% in rural areas, and the remaining in the urban-rural integration zone. The participants were divided into groups based on cigarette use, alcohol consumption, nap duration, and sleep duration, as shown in Table 1.

**Table 1:** Baseline population characteristics and prevalence in the grouped population.

**Characteristics**	**Total** **participants** **(%)**	**Cognitive** **impairment**	**Prevalence** **(%)**	**95% CI**
**Total**	3,768 (100%)	838	22.24	20.94–23.60
**Age groups**				
60–70 years	2,638 (70.0%)	555	21.04	19.52–22.64
70–80 years	983 (26.1%)	235	23.91	21.34–26.68
>80 years	147 (3.9%)	48	32.65	25.51–40.70
**Gender**				
Male	2,451 (65.0%)	534	21.79	20.20–23.47
Female	1,317 (35.0%)	304	23.08	20.88–25.44
**Marital status**				
Married with spouse present	3,054 (81.1%)	635	20.79	19.39–22.27
Separated/divorced/ widowed/ never married	714 (18.9%)	203	28.43	25.24–31.86
**Residence**				
Urban	1,153 (30.8%)	179	15.52	13.55–17.73
Urban-Rural Integration Zone	320 (8.5%)	61	19.06	15.11–23.76
Rural	2,275 (60.7%)	593	26.07	24.30–27.91
**Cigarette consumption**				
Yes	2,061 (54.7%)	471	22.85	21.09–24.72
No	1,703 (45.3%)	367	21.55	19.66–23.57
**Alcohol consumption**				
More than Once a Month	1,227 (32.6%)	256	20.86	18.68–23.23
Less than Once a Month	337 (9.0%)	57	16.91	13.27–21.32
None of These	2,200 (58.4%)	525	23.86	22.13–25.69
**Nap duration**				
≤ 30 min	1,960 (52.1%)	428	21.84	20.06–23.72
30–60 min	1,067 (28.3%)	205	19.21	16.96–21.69
60–90 min	233 (6.2%)	58	24.89	19.74–30.88
≥ 90 min	504 (13.4%)	147	29.17	25.35–33.30
**Sleep duration**				
≤ 6 h	2,087 (55.4%)	459	21.99	20.27–23.82
6–8 h	1,409 (37.4%)	286	20.30	18.28–22.48
≥8 h	268 (7.1%)	93	34.70	29.21–40.62
**Depression**				
Yes	1,206 (32.0%)	355	29.44	26.93–32.07
No	2,561 (68.0%)	483	18.86	17.39–20.42
**Hypertension**				
Yes	435 (11.5%)	106	24.37	20.55–28.64
No	3,333 (88.5%)	732	21.96	20.59–23.40
**Diabetes**				
Yes	214 (6.4%)	51	23.83	18.57–30.03
No	3,135 (93.6%)	713	22.74	21.31–24.24
**Arthritis**				
Yes	259 (6.9%)	64	24.71	19.82–30.36
No	3,509 (93.1%)	774	22.06	20.72–23.46
**Digestive diseases**				
Yes	280 (7.4%)	56	20.00	15.70–25.12
No	3,488 (92.6%)	782	22.42	21.07–23.83
**Kidney diseases**				
Yes	174 (4.6%)	42	24.14	18.32–31.10
No	3,594 (95.4%)	796	22.15	20.82–23.54
**Liver diseases**				
Yes	150 (4.0%)	23	15.33	10.37–22.08
No	3,618 (96.0%)	815	22.53	21.19–23.92
**Stroke**				
Yes	233 (6.3%)	53	22.75	17.79–28.60
No	3,441 (93.7%)	767	22.29	20.93–23.71

CI, Confidence Interval.

### Age- and gender-specific prevalence

**Figure 1:**
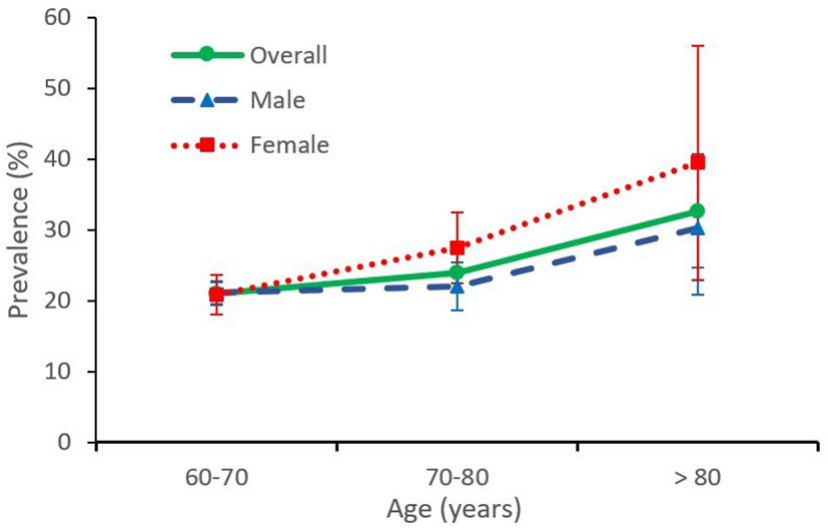
Age and gender-specific prevalence of mild cognitive impairment. The prevalences of MCI in females are higher than those in males in the 70–79 years old group and the >80 years old group. Error bars represent 95% CIs.

As shown in Figure 1, the prevalence of cognitive impairment increased with age. The prevalence rates were 21.04% (95% CI, 19.52–22.64) in people aged 60–69 years, 23.91% (95% CI, 21.34–26.68) in people aged 70–79 years, and 32.65% (95% CI, 25.51–40.70) in people aged over 80 years. No significant difference in prevalence was found between female and male participants aged 60–69 years (20.79% vs. 21.17%). However, the prevalence of cognitive impairments in females was 1.25 and 1.30 times that in males in participants aged 70–79 years (27.46 vs. 21.98%) and in participants aged >80 years (39.47 vs. 30.27%), respectively.

### Gender-specific prevalence across regions

**Figure 2:**
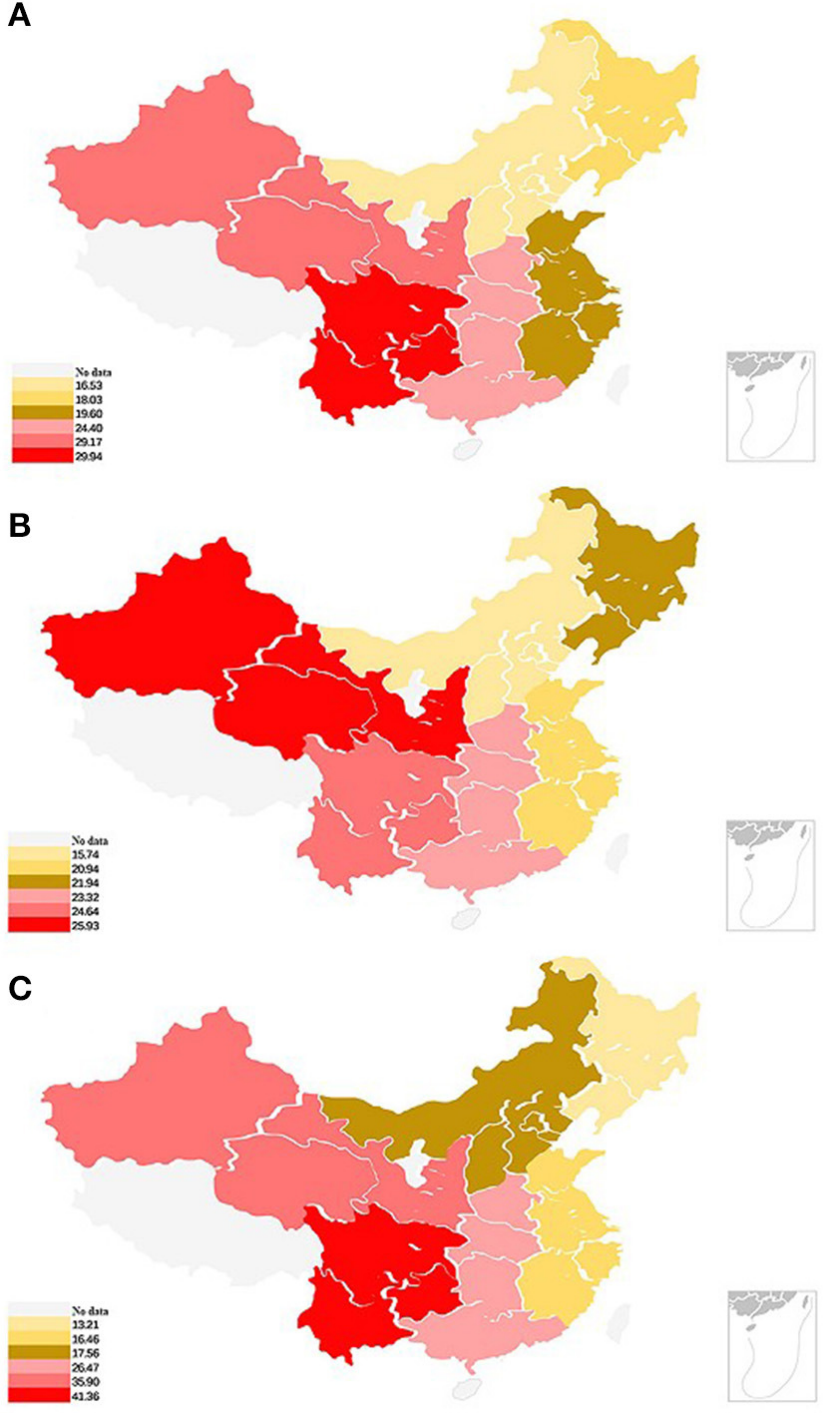
Gender-specific prevalence of cognitive impairment in different regions. The prevalence overall **(A)**, in males **(B)** and in females **(C)** of cognitive impairment among the elderly population were calculated, and living localities of the participants were also categorized into six regions. People living in the south-west region have the highest prevalence, while people living in the north region have the lowest prevalence. North region including Shanxi, Hebei, Beijing, Tianjin, and Inner Mongolia. North-East region including Jilin, Liaoning, and Heilongjiang. East region including Jiangsu, Fujian, Shanghai, Shandong, Zhejiang, Jiangxi, and Anhui. North-West region including Qinghai, Shanxi, Xinjiang, and Gansu), South-West region including Sichuan, Chongqing, Yunnan, and Guizhou. and South-Central region including Hunan, Henan, Guangdong, Hubei, and Guangxi.

Gender-specific prevalence in different regions is displayed in Figure 2. The highest prevalence of cognitive impairments was found in the southwest region (29.94%); and the lowest, in the north region (16.53%). Difference in prevalence was found between males and females across regions. The prevalence of cognitive impairments in females was 1.68 and 1.38 times that in males in the southwest region (41.36 vs. 24.64%) and northwest region (35.90 vs. 25.93%), respectively.

### Associated factors of cognitive impairments

As shown in Table 2, results from univariate logistic regression indicated increased risk of cognitive impairments in participants aged > 80 years compared with participants aged 60–70 years (odds ratio, OR = 1.82, 95% CI, 1.27–2.60; *p* < 0.001), participants that were not married or cohabitating compared with married with spouse present (OR = 1.51, 95% CI, 1.26–1.82; *p* < 0.001), participants with daytime napping of ≥90 min compared those with 30–60 min (OR = 1.73, 95% CI, 1.35–2.21; *p* < 0.001), participants with sleeping duration at night of ≥8 h compared those with 6–8 h (OR = 2.09, 95% CI, 1.57–2.77; *p* < 0.001), and participants with depression compared with those without depression (OR = 1.79, 95% CI, 1.53–2.10; *p* < 0.001). Meanwhile, a lower risk of cognitive impairments were found in participants living in urban areas compared with those living in rural areas (OR = 0.52, 95% CI, 0.43–0.63; *p* < 0.001), participants with less alcohol consumption compared with those who never consumed alcohol (OR = 0.65, 95% CI, 0.48–0.88; *p* = 0.005) or participants with liver diseases than those without liver diseases (OR = 0.62, 95% CI, 0.40–0.98; *p* = 0.04).

**Table 2:** The associated factors and adjusted ORs.

**Characteristics**	**Crude OR** **(95% CI)**	***p*** **-value**	**Adjusted** **OR (95%** **CI)**	***p*** **-value**
**Age groups**				
60–70 years	1.00 (reference)	-	1.00 (reference)	-
70–80 years	1.18 (0.99–1.40)	0.063	1.08 (0.90–1.30)	0.398
>80 years	1.82 (1.27–2.60)	<0.001	1.61 (1.11–2.34)	0.013
**Gender**				
Male	1.00 (reference)	-	-	-
Female	1.08 (1.92–2.60)	0.362	-	-
**Marital status**				
Married with spouse present	1.00 (reference)	-	1.00 (reference)	-
Others	1.51 (1.26–1.82)	<0.001	1.39 (1.15–1.70)	0.001
**Residence**				
Central of City/Town	0.52 (0.43–0.63)	<0.001	0.57 (0.47–0.69)	<0.001
Urban-Rural Integration Zone	0.67 (0.50–0.90)	0.007	0.75 (0.56–1.01)	0.061
Rural	1.00 (reference)	-	1.00 (reference)	-
**Cigarette consumption**				
Yes	1.00 (reference)	-	-	-
No	0.93 (0.79–1.08)	0.339	-	-
**Alcohol consumption**				
More than Once a Month	0.84 (0.71–0.99)	0.045	0.89 (0.74–1.06)	0.175
Less than Once a Month	0.65 (0.48–0.88)	0.005	0.69 (0.51–0.94)	0.02
Never	1.00 (reference)	-	1.00 (reference)	-
**Nap duration**				
≤ 30 min	1.17 (0.98–1.41)	0.09	1.13 (0.93–1.36)	0.225
30–60 min	1.00 (reference)	-	1.00 (reference)	-
60–90 min	1.39 (0.99–1.95)	0.051	1.36 (0.97–1.92)	0.078
≥90 min	1.73 (1.35–2.21)	<0.001	1.54 (1.20–1.98)	0.001
**Sleep duration**				
≤ 6 h	1.11 (0.94–1.31)	0.23	1.02 (0.85–1.21)	0.87
6–8 h	1.00 (reference)	-	1.00 (reference)	-
≥8 h	2.09 (1.57–2.77)	<0.001	1.73 (1.29–2.31)	<0.001
**Depression**				
Yes	1.79 (1.53–2.10)	<0.001	1.67 (1.41–1.97)	<0.001
No	1.00 (reference)	-	1.00 (reference)	-
**Self-reported hypertension**				
Yes	1.14 (0.91–1.45)	0.257	-	-
No	1.00 (reference)	-	-	-
**Diabetes**				
Yes	1.06 (0.77–1.47)	0.714	-	-
No	1.00 (reference)	-	-	-
**Arthritis**				
Yes	1.16 (0.86–1.56)	0.322	-	-
No	1.00 (reference)	-	-	-
**Digestive diseases**				
Yes	0.87 (0.64–1.17)	0.349	-	-
No	1.00 (reference)	-	-	-
**Kidney diseases**				
Yes	1.12 (0.78–1.60)	0.538	-	-
No	1.00 (reference)	-	-	-
**Liver diseases**				
Yes	0.62 (0.40–0.98)	0.04	0.65 (0.41–1.03)	0.064
No	1.00 (reference)	-	1.00 (reference)	-
**Stroke**				
Yes	0.97 (0.71–1.34)	0.871	-	-
No	1.00 (reference)	-	-	-

Logistic regression was adopted to identify the associated independent factors of cognitive impairment. All plausible variables with P < 0.05 in univariate testing were subjected to further multivariate testing. The crude ORs were calculated in univariate regression, and the adjusted ORs were recorded using multivariate regression. OR, Odds Ratio; CI, Confidence Interval.

Multivariate logistic regression models were used in evaluating risk factors for cognitive impairment. The final multiple logistic regression model included age, marital status, residence, alcohol consumption, nap duration, sleep duration, depression and liver disease. The prevalence of cognitive impairment was higher in participants who were not married or were not living with spouse compared with those married living with their spouses (OR = 1.39, 95% CI, 1.15–1.70; *p* = 0.001) and participants with nap duration of ≥90 min compared with those of nap duration of 30–60 min (OR = 1.54, 95% CI, 1.20–1.98; *p* = 0.001), participants with sleep duration of ≥8 h compared with those with sleep duration of 6–8 h (OR = 1.73, 95% CI, 1.29–2.31; *p* < 0.001), and participants with depression compared with those without depression (OR = 1.67, 95% CI, 1.41–1.97; *p* < 0.001). Notably, a decreased risk of cognitive impairment was observed in participants living in urban areas compared with those living in rural areas (OR = 0.57, 95% CI, 0.47–0.69; *p* < 0.001) and in participants with a low-alcohol consumption (less than once a month) compared with those who never consumed alcohol (OR = 0.69, 95% CI, 0.51–0.94; *p* = 0.02), indicating low risk of cognitive decline.

## Discussion

In this nationwide cross-sectional study, we estimated the prevalence and associated risk factors for cognitive impairments in the Chinese elderly population. The highest prevalence of cognitive impairments was found in the southwest region of China, and the lowest was found in the north region, indicating regional differences. Several associated risk factors, including marital status, urban or rural residence, sleep and nap durations, depression, and alcohol consumption, were identified.

With regard to aging, the global elderly population is growing rapidly, especially in mainland China. According to China's Seventh National Population Census in 2020, the number of elderly people over 60 years has reached 264.02 million, accounting for 18.7% of the total population (23). Rapid growth in the elderly population has stimulated interest in elucidating the causes of cognitive impairments and strategies for preventing them. Lu (16) conducted a study in Ji County of Tianjing (a rural area of northern China) and suggested that the prevalence of cognitive impairment is 38.3% (27.8% MCI and 10.5% dementia) in the overall population aged 60 years or older. After studying 96 sites from 12 provinces, Jia (6) suggested that the prevalence of cognitive impairment was 21.5% (15.5% MCI and 6.0% dementia) in the overall population aged 60 years or older in 2015–2018. A meta-analysis comprising 48 studies with 102,906 participants reported that the overall prevalence of MCI is 14.71% in Chinese people aged 60 years or older (24). A meta-analysis comprising 96 studies reported that the overall prevalence of dementia is 5.3% in Chinese people aged 60 years or older (25). The CHARLS national survey covers 150 counties or districts (a total of 450 villages or resident committees) from 28 provinces from 2018 to 2019. These data were used in estimating the nationwide prevalence of cognitive impairments in the Chinese elderly population. The present study estimated the nationwide prevalence of cognitive impairments (MCI and dementia) at 22.24% in participants aged 60 years and older, revealing prevalence similar to that presented by most reports from other populations in China (6, 24, 25), the United States (16.0%−22.2%) (26), and South Korea (24.1%) (27).

This study found an apparent geographical variation in the prevalence of cognitive impairments in China. As shown in Figure 2, the results of prevalence distribution suggests that the incidence in western China (southwest and northwest regions) was the highest, and the prevalence in the southwest region was 1.81 times that in the north region. A meta-analysis reported that the pooled prevalence of dementia was the highest in western China (9.6%), intermediate in northern China (5.4%), and lowest in central China (3.8%) and south China (3.7%) (25). Another meta-analysis reported that the pooled prevalence of MCI was higher in western China (14.33%) than in eastern China (13.41%) (24). Dietary differences may contribute to this discrepancy. The daily diet of participants living in the north regions contains considerable amounts of milk, dairy products, and flour-based food, whereas participants living in the southwest regions consume more fruits and rice-based diet; dairy products have been confirmed to have a protective effect against cognitive impairment (28). Other differences may be attributed to the uneven economic, educational development, and different living habits across regions in China.

The identification of specific risk factors is crucial for the prevention of cognitive impairments. The prevalence of cognitive impairment was higher among elderly people, females, people who are not married or cohabitating, and people living in rural areas or western China, consistent with the findings of some previous studies (6–8, 10, 24). The present study provides further evidence in support of these risk factors. The results showed that depression is associated with the high prevalence of MCI in the Chinese elderly population, consistent with previous findings (14, 23). Jia et al. (6) showed high prevalence of cognitive impairment in the Chinese elderly population with hypertension (odds ratio: dementia = 1.86; MCI = 1.62), hyperlipidemia (dementia = 1.87; MCI = 1.29), diabetes (dementia = 2.14; MCI = 1.44), heart disease (dementia = 1.98; MCI = 1.17), and cerebrovascular disease (dementia = 5.44; MCI = 1.49), but the above risk factors were not found in the present study. Medical histories were mainly based on the self-reports of the respondents, which may lead to deviation from the present results.

The present study suggested that some other risk factors are modifiable, including nap duration, sleep duration, and alcohol consumption, which are rarely explored. Sleep disturbances are common in the elderly, and approximately 50% of people aged over 65 years reported a chronic sleep complaint (29). Many studies have examined the associations between sleep duration and cognitive impairment. Moreover, The results also suggest that long durations of napping (≥90 min) and long sleeping (≥8 h) are associated with high prevalence of cognitive impairment and an afternoon nap duration of 30–60 min and night sleep of 6–8 h are associated with enhanced cognitive function. The exact biological mechanisms linking excessive sleep nap duration to cognitive impairment remain unclear (30). Further studies are needed to examine whether excessive sleeping or napping is a subtle marker of cognitive impairment in otherwise healthy elderly individuals.

Epidemiological studies have indicated that excessive alcohol consumption can induce cognitive impairments, whereas moderate consumption may reduce the risks for cognitive impairment in the elderly (31, 32). The present study suggested a similar result, that is, consuming alcohol less than once a month is associated with a lower prevalence of cognitive impairment (OR = 0.65, *p* = 0.005) than that in participant who never consumed alcohol. The potential factors linking low alcohol consumption to cognitive function have been attributed to flavonoids or other antioxidants, which may reduce the risk of cognitive impairment (33).

The present study has some limitations. First, we did not classify MCI and dementia. Clinical diagnosis based on the MMSE, the Montreal Cognitive Assessment, Clinical Dementia Rating score, magnetic resonance imaging, or computed tomography is necessary for MCI and dementia (6). MMSE as a single diagnostic tool is insufficient to diagnose MCI and dementia, and different diagnostic confirmation tools might result in different prevalence rates. Second, gender selection bias was found; more male participants were selected. Third, relying on the participants' self-reporting, the medical histories, nap durations, and sleep durations might not be accurate measures and might generate bias. In the evaluation of covariates, we did not employ objective monitoring devices because collecting data from a large cohort is difficult. Additionally, this cross-sectional study cannot establish causal relationships between the identified associated factors and cognitive impairment, and future longitudinal studies are needed to clarify these associations.

## Conclusion

In this nationwide cross-sectional study, the overall prevalence of cognitive impairments was 22.24% in the participants aged 60 years or over. The prevalence varied among age groups, living areas, regions and between genders. The results revealed that the modifiable risk factors for cognitive impairment were as follows: not married or cohabitating, rural residence, long duration of napping (≥90 min), long duration of sleep (≥8 h), and depression. Thus, preventive strategies for cognitive impairment are needed to improve cognitive function.

## Data availability statement

The original contributions presented in the study are included in the article/Supplementary material, further inquiries can be directed to the corresponding author.

## Ethics statement

The CHARLS study was approved by Research Ethics Committees of Peking University (IRB00001052-13074). The patients/participants provided their written informed consent to participate in this study.

## Author contributions

FQ and ML wrote the manuscript and participated in all aspects of this research. XC edited, reviewed, and supervised this research. YX, NZ, YD, and WK reviewed the final article. All authors read and approved the final manuscript.

## Funding

This work was supported by the National Natural Science Foundation of China (82071494 and 81871043) and National Key R&D Program of China (2022YFC2009902/2022YFC2009900).

## Conflict of interest

The authors declare that the research was conducted in the absence of any commercial or financial relationships that could be construed as a potential conflict of interest.

## Publisher's note

All claims expressed in this article are solely those of the authors and do not necessarily represent those of their affiliated organizations, or those of the publisher, the editors and the reviewers. Any product that may be evaluated in this article, or claim that may be made by its manufacturer, is not guaranteed or endorsed by the publisher.

## Supplementary material

Supplementary Figure 1 Flowchart of data cleansing. MMSE: Mini-Mental State Examination.

Supplementary Figure 2 Matrix of the Spearman's correlation coefficient.

## Abbreviations

CESD-10: Center for Epidemiological Studies Depression Scale-10
CHARLS: China Health and Retirement Longitudinal Study
MCI: Mild cognitive impairment
MMSE: Mini-Mental State Examination.
