# Supplementary figures and images for: Prevalence and associated factors of cognitive impairment among the elderly population: A nationwide cross-sectional study in China
Source: Front Public Health. 2022 Nov 17;10:1032666. doi: 10.3389/fpubh.2022.1032666 (PMC9713248; doi:10.3389/fpubh.2022.1032666)

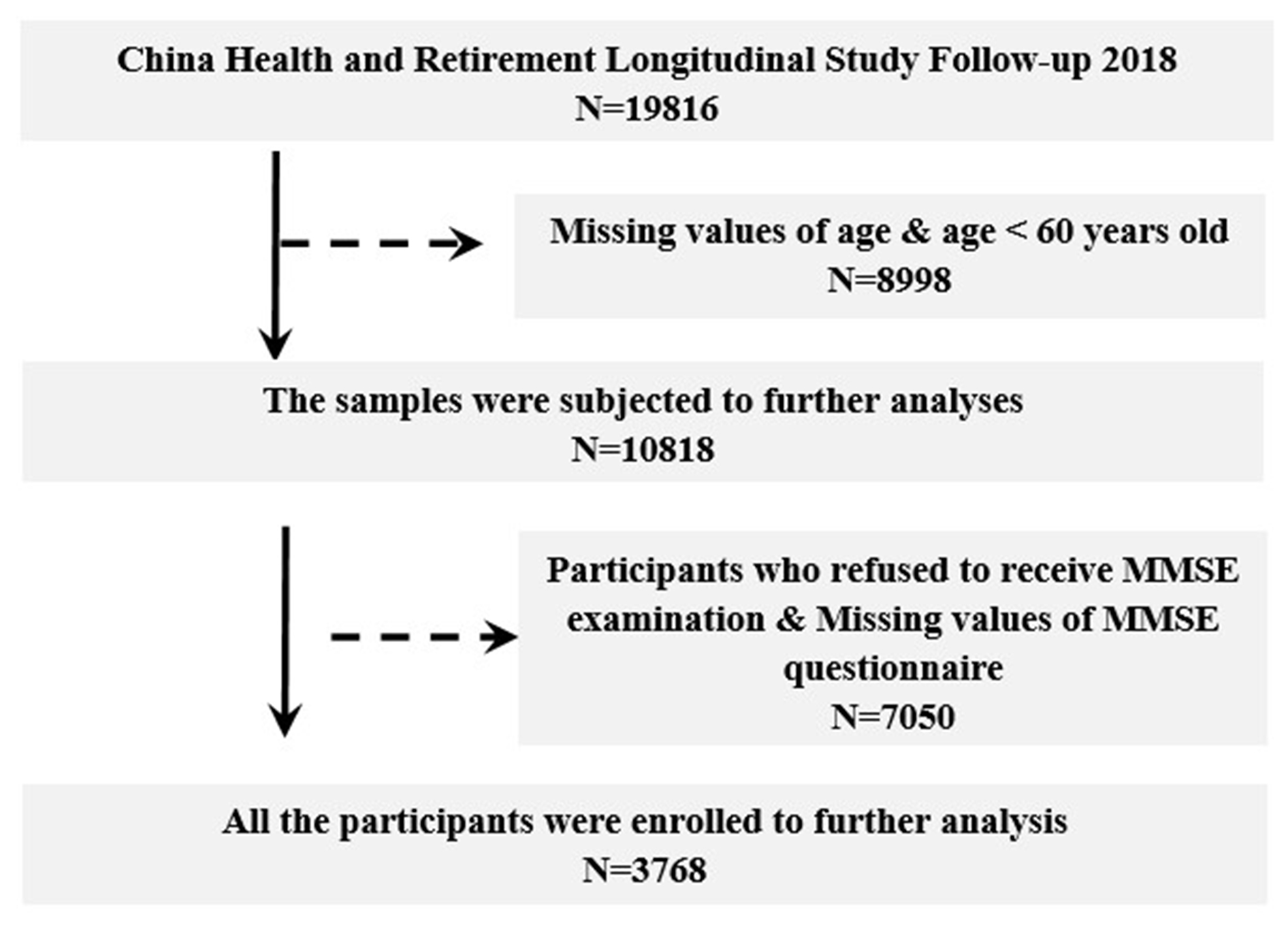

Supplement: Supplementary Figure 1 — Flowchart of data cleansing. MMSE: Mini-Mental State Examination. [file Image_1.JPEG]

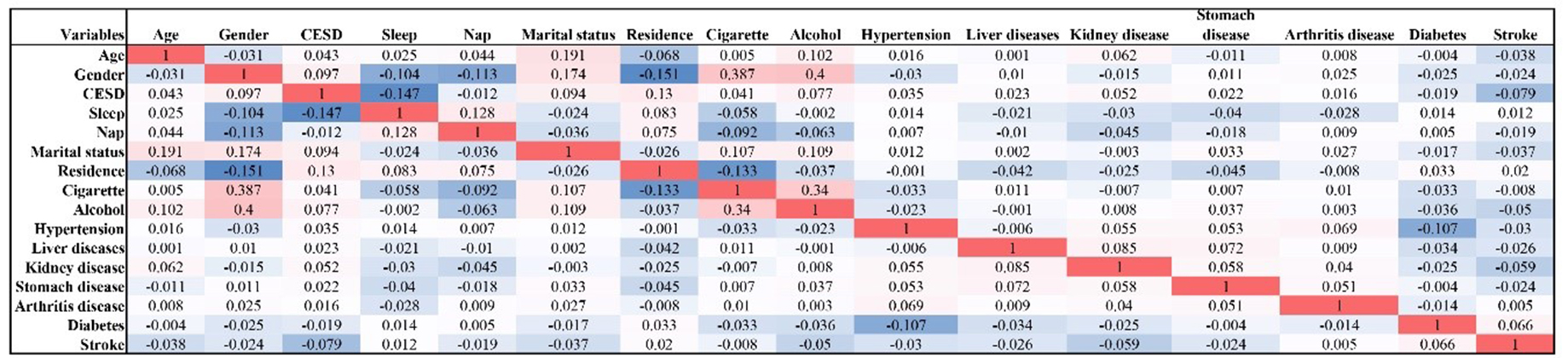

Supplement: Supplementary Figure 2 — Matrix of the Spearman's correlation coefficient. [file Image_2.JPEG]
